# Antitumor Effect of Demethylzeylasteral (T-96) on Triple-Negative Breast Cancer via LSD1-Mediate Epigenetic Mechanisms

**DOI:** 10.1155/2022/2522597

**Published:** 2022-10-12

**Authors:** Zhengjie Shen, Yongjuan Gu, Ruiyang Jiang, Heya Qian, Siyuan Li, Lixian Xu, Wenzhe Gu, Yun Zuo

**Affiliations:** ^1^The Affiliated Zhangjiagang Hospital of Soochow University, Zhangjiagang 215600, China; ^2^Zhangjiagang TCM Hospital Affiliated to Nanjing University of Chinese Medicine, Zhangjiagang 215600, China; ^3^Jiangsu Collaborative Innovation Center of Traditional Chinese Medicine Prevention and Treatment of Tumor, Nanjing 210023, China; ^4^Nanjing University of Chinese Medicine, Nanjing 210023, China

## Abstract

*Background and Purpose*. Breast cancer ranks first in the incidence of female tumors. Triple-negative breast cancer (TNBC), one type of breast cancer, is more aggressive and has a worse prognosis. Demethylzeylasteral (T-96) is isolated from *Tripterygium wilfordii* Hook F. Our previous study found that T96 could inhibit TNBC invasion via suppressing the canonical and noncanonical TGF-*β* signaling pathways. However, the antitumor effects and mechanisms of T-96 on TNBC have not been studied. This study is aimed at investigating the antitumor effect and mechanism of T-96 on breast cancer. *Experimental approach*. MTT assay, Live and Dead cell assay, and TUNEL were used to observe the antitumor effect of breast cancer cells treated with T-96. siRNA of LSD1, Co-IP, and molecular docking were used to explore the direct target and mechanism of T-96. Subcutaneous murine xenograft models were used to detect the efficacy of T-96 antitumor activity in vivo. *Key Results*. T-96 was more susceptible to inducing the apoptosis of highly metastatic TNBC cell lines (SUM-1315). An abnormal level of histone methylation is a crucial characteristic of metastatic cancer cells. LSD1 is a histone demethylase. We found that T-96 could significantly decrease the protein expression of LSD1, increase its target protein PTEN expression and enhance histone methylation. T-96 could also down-regulate the PI3K/AKT signaling pathway, which could be blocked by PTEN. Knockdown of LSD1 by siRNA blocked the pharmacological activity of T-96. And the molecular docking predicted T-96 processed affinity toward LSD1 through hydrogen bonding. Finally, T-96 was evaluated in a murine xenograft model of SUM-1315 cells. And T-96 could significantly inhibit tumor growth without showing marked toxicity. *Conclusions & Implications*. The results illustrated that T-96 exerted antitumor activity in highly metastatic TNBC by inactivating the LSD1 function.

## 1. Introduction

According to the global cancer statistics 2020, breast cancer has been the most commonly diagnosed cancer and the leading cause of cancer death in women. It is estimated that in 2020 there would be 2.26 million new cases (11.7% of total cases) and 684,996 new deaths (6.9% of total cases) in 36 cancers [1]. Triple-negative breast cancer (TNBC) accounting for approximately 15% of invasive breast cancers, is the most aggressive subtype, which has the characteristic of loss of expression of estrogen receptor (ER) and progesterone receptor (PR) and does not have amplification of the epidermal growth factor receptor 2 (ERBB2). Because of the absence of antihormone and anti-ERBB2 targeted therapies, chemotherapy has been the first-line therapeutic option for TNBC patients. But the clinical response to chemotherapy is limited and associated with toxicity [2, 3]. Therefore, developing novel drugs and therapies is essential for TNBC.

Epigenetics, which involves noncoding-RNAs, histone modifications, and DNA methylation, is the study of potentially heritable changes in gene expression without changes in the DNA sequences or genotype. [4–6] Histone methylation, a process in that methyl group is transferred to amino acids of histone proteins, has been associated with cancer, cardiovascular disease, and Alzheimer's disease [7–9]. Histone methyltransferases and histone demethylases, which methylate and demethylate protein lysine and arginine residues, have crucial roles in the control of histone methylation regulation [10, 11]. So, this family of enzymes plays key roles in normal physiology and human diseases and could be novel, chemically tractable potential therapeutically targets for drug discovery.

Lysine (K)-specific demethylase 1A (LSD1) is the first discovered histone lysine demethylase and is encoded by the *KDM1A* gene [12]. LSD1, which is a flavin-dependent monoamine oxidase, can demethylate mono- and di-methyl groups on the H3K4 for suppressing gene expression, or convert di-methylated H3K9 to mono- and unmethylated H3K9 for increasing gene expression [13, 14]. This protein matters in embryonic stem cell self-renewal and tissue-specific differentiation, which as well as regulates other pathological processes [15, 16]. LSD1 is also thought to have a vital function in various tumor biological processes, such as proliferation, cell survival, and epithelial-to-mesenchymal transition (EMT). And the higher expression of LSD1 indicates poorer outcomes [17–19]. Therefore, the inhibition of LSD1 may be a promising drug target for cancer therapy [20, 21].

*Tripterygium wilfordii* Hook F. (TWHF) is a traditional Chinese herbal, which is widely distributed in China, Japan, and Korea. Demethylzeylasteral (T-96), the most important constituent of TWHF, is extracted from the TWHF whole plant or peeled wood parts [22]. And some research has demonstrated that T-96 has an obvious immunomodulatory effect and antitumor activity, such as suppressing inflammation, inhibiting tumor growth, enhancing chemosensitivity, etc. [23–25]. It has been reported that T-96 could also suppress the tumorigenicity induced by liver cancer stem cells by inhibiting H3 histone lactylation, which indicates that the antitumor effects of T-96 are related to epigenetic molecules [26]. So, we hypothesize that T-96 has an antitumor effect on TNBC cells through LSD1-mediated histone modification. In the previous study, we found that T-96 could reverse EMT and inhibit the invasion of TNBC cells by suppressing the TGF-*β* signaling pathway [27]. However, its antitumor effects and mechanisms have not been thoroughly studied. In this study, we investigated T-96's antitumor activity on two breast cancer cell lines, SUM-1315 cell line, a highly metastatic and *BRCA1* mutant TNBC cell line, and MCF-7 cell line, a lowly metastatic and ER-positive breast cancer cell line. And we also explored whether T-96's anti-tumor mechanism is through LSD1-mediated histone modification.

## 2. Materials and Methods

### 2.1. Reagents and Materials

Dulbecco's modified Eagle's medium (DMEM), Penicillin-Streptomycin (10,000 U/mL), fetal bovine serum (FBS), and L-Glutamine (200 mM) were obtained from Thermo Fisher Scientific Inc., US. MTT and DMSO were obtained from Sigma Chemical Co., US. All antibodies were obtained from Abcam Co., US. T-96 was purchased from BioBioPha Co., China.

### 2.2. Cell Lines and Cell Culture

Human breast cancer cell lines SUM-1315 and MCF-7 were cultured in DMEM supplemented with 10% FBS, Penicillin-Streptomycin (100 U/mL), and L-Glutamine (2 mM) at 37°C with 5% CO_2_ in a humidified incubator. Cells were digested with 0.5 mL 0.25% trypsin for 1-3 minutes at 37°C. And 2 mL complete medium was added to neutralize the trypsin. The cells were used during their logarithmic growth phase. [28]

### 2.3. Cell Viability Assay

Cell viability was detected by 3-(4,5-dimethylthiazol-2-yl)-2,5-diphenyl tetrazolium bromide (MTT) assay. First, we seeded the cells at a concentration of 5×103 cells/well in a 96-well plate for 24 h. T-96 was dissolved in DMSO and prepared to a parent solution concentration of 100 mM. And then, the cells were grown in different concentrations of T-96 (1, 2, 4, 8, 12 *μ*M) for 24 h or 48 h. Cisplatin (CP, 8 *μ*M) was used as a positive control group. After being treated with T-96 or CP for 24 h or 48 h, the 10 *μ*L MTT solutions (5 mg/ml) was added to each well and incubated for 4 h. 100 *μ*L DMSO was added to each well to solute the crystal. Absorbance was measured by the plate reader at a wavelength of 492 nm. [29]

Live and Dead cell assay was also used to detect the cell viability. The Live and Dead cell assay staining solution is a mixture of two fluorescent dyes that differentially label live and dead cells. The Live cell dye labels intact, viable cells green. The Dead cell dye labels cells with compromised plasma membranes red. A Live and Dead staining kit (Yeasen, China) was used to assess the antitumor activity of T-96 on SUM-1315 cells. After being treated with or without T-96 for 48 h, the cells were collected through a cell scraper. Cells were washed, and after centrifugation, a cell suspension of density 10^5^ cells/mL was made with 1 × Assay Buffer. Add 100 *μ*L staining reagent to 200 *μ*L cell suspension, incubated for 15 min at 37°C, and then be observed under fluorescence microscopy (Nikon, Japan).

### 2.4. Transient Transfection

The cells were inoculated in six-well plates. LSD1-specific siRNA (sc-60970, Santa Cruz, US) or nontarget-specific control siRNA (sc-37007, Santa Cruz, US) was transiently transfected by Invitrogen™ Lipofectamine 3000 transfection reagent, according to instructions provided with the reagent. [30] After that, the cells were treated with T-96 and cultured for 48 h, and the proteins were harvested for subsequent studies.

### 2.5. Coimmunoprecipitation (coIP) Assay

In the standard Co-IP assay, cells were lysed in RIPA buffer (Beyotime, China). The cell lysates were clarified by centrifugation at 14,000 × g for 10 minutes, and the supernatant was collected. After removing the bottom precipitate, 2 *μ*g of the primary antibody was added to 1 mg of clarified total cell lysates and incubated overnight at 4°C. The next day, Protein A-agarose beads (Santa Cruz, US) were added and incubated for 2 h. Then, the beads were washed three times with ice-cold RIPA buffer, followed by adding 1× SDS loading buffer to resuspend. After microcentrifugation for 30 s, the sample is heated to 96°C for 10 min and centrifuged for 1 min at 14,000 × g. [31, 32]

### 2.6. Extract the Nuclear Protein

The nuclear protein was prepared in accordance with the protocol from a Nuclear Extraction Kit (Beyotime, China). Remove the growth medium and wash cells with PBS, after removing the PBS, scrap off cells with a cell scraper. Cells were harvested by centrifugation. Vortex for 5 seconds, the cell precipitate was completely suspended and dispersed. Put the sample on ice for 5 min. Cytoplasmic protein extraction reagent B was added, vortexed, and centrifuged, and the supernatant was removed to a new EP tube. For precipitation, the residual supernatant was completely aspirated and a nuclear protein extraction reagent supplemented with PMSF was added. After vortex and centrifugation, the supernatant was taken, which was the extracted nuclear protein.

### 2.7. Western Blot Analysis

After treatment with various concentrations of T-96 for 48 h, total cellular proteins were extracted by RIPA buffer, electrophoresed on 12% SDS-PAGE, and transferred to PVDF membrane by semidry apparatus for 35 min. The membrane was blocked and incubated with the primary antibody at recommended concentrations overnight at 4°C. Next day, the membrane was washed with PBS three times, and incubated with the secondary antibody for 1 h, and then the membrane was incubated with the ECL. Finally, the signals were detected by Tanon Western Blot System (Shanghai, China). [33]

### 2.8. Murine Models

All murine experiments were conducted under protocols approved by the Animal Care and Use Committee of Jiangsu Province Academy of Traditional Chinese Medicine. The nude mice were raised in an air-conditioned pathogen-free environment. SUM-1315 cells (1 × 10^7^ cells) were injected subcutaneously into the right flank of the nude mice. When tumors became palpable, the mice were randomly divided into two groups and treated with T-96 (5 mg/kg) or vehicle (1% DMSO) in normal saline for 25 days. The body weight and tumor size were measured every 5 days. On the 25th day, all mice were sacrificed; the tumors and tissue were excised, and frozen inside a -80°C refrigerator for the next experiments.

### 2.9. Immunohistochemistry Assay

Tumor tissues were formalin-fixed, paraffin-embedded, and sectioned at 5 *μ*m thick. Sections were deparaffinated and rehydrated, and endogenous peroxidase activity was blocked with 3.0% H_2_O_2_. Then, Sections were incubated overnight with the primary antibody followed by the secondary antibody for 30 minutes. Staining was visualized using the DAB Kit (Keygentec, China). [34]

### 2.10. Terminal Deoxynucleotidyl Transferase dUTP Nick End Labeling (TUNEL) Assay

TUNEL staining of paraffin-embedded tumor sections was performed according to protocols provided by the manufacturer (Roche Diagnostics, Mannheim Germany). Images were acquired with Nikon Microscope.

### 2.11. Immunofluorescent Assay

Tumor tissues were sectioned and analyzed by immunofluorescence as described [35]. Images were acquired with Nikon Microscope.

### 2.12. Docking Molecules

T-96 was drawn with Chemdraw 2014 and opened in Maestro 10.2. Then, the ligands were processed with the Ligand preparation protocol in Maestro 10.2. And LSD1 (PDB id: 2Z5U) was downloaded from PDB (http://www.rcsb.org/pdb) and opened by Maestro 10.2. The Glide docking protocol was adopted for the docking studies.

### 2.13. Statistical Analysis

Data were presented as mean ± SD and analyzed by SPSS 15.0 software, and all the raw data was carried out on the *T*-test. *P* < 0.05 was considered statistically significant.

## 3. Result

### 3.1. T-96 Selectively Kills Highly Metastatic Breast Cancer Cell Line

**Figure 1:**
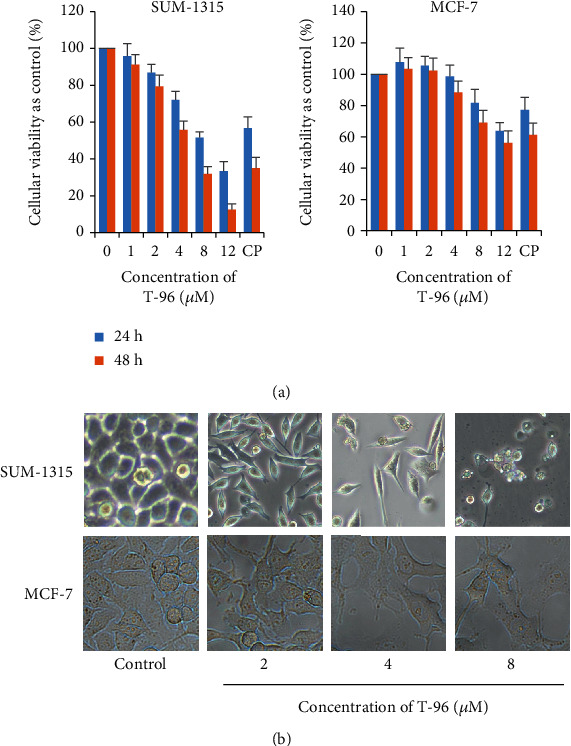
T-96 selectively killed highly metastatic breast cancer cell lines. (a) T-96 inhibited cancer cell growth; (b) microscopy was used to observe the cell morphology.

In Figure 1(a), MTT assays showed that T-96 induced cytotoxicity more effectively in the highly metastatic TNBC cell line (SUM-1315) than in the lowly metastatic breast cancer cell line (MCF-7). T-96 caused morphological changes in these breast cancer cells tested (Figure 1(b)). The SUM-1315 cell volume decreased, the intercellular junctions disappeared, and some cells shrank and became round. Significant changes were observed in the high-dose group (8 *μ*M). Treatment of the SUM-1315 cell line with T-96 resulted in a significantly higher number of cell apoptosis compared with the MCF-7 cell line. So, compared with the lowly metastatic breast cancer cell line, T-96 displayed a preferential antiproliferative activity against the highly metastatic breast cancer cell line (SUM-1315). Thus, SUM-1315 was chosen as a model for further investigation of the antitumor activity and the underlying mechanisms of T-96.

### 3.2. T-96 Induces Apoptosis in SUM-1315 Cells

**Figure 2:**
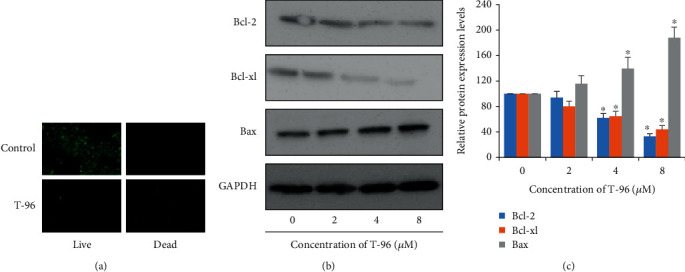
T-96 induced apoptosis in the highly metastatic breast cancer cell SUM-1315. (a) Analysis of the dead and live cell by LIVE/DEAD™ Viability; (b) and (c) Western blot was used to measure Bcl-2, Bcl-xl, and Bax protein expressions. Values are statistically significant at ^∗^*P* < 0.05.

Live and Dead assay and Western Blotting assay demonstrated that T-96 reduced the viability of SUM-1315 cells in a concentration-dependent manner via apoptosis in Figure 2. By Live and Dead assay, compared with the control group, the number of green fluorescent cells significantly decreased and red fluorescence increased after T-96 treatment. We found that T-96 drastically induced cell death (Figure 2(a)). And next, the Western Blotting assay was used to detect apoptosis-related proteins expression, and we found that Bcl-2 and Bcl-xl, two antiapoptotic proteins, were significantly decreased in the treatment group. But Bax, a proapoptotic protein, was significantly increased in the treatment group (Figures 2(b) and 2(c)). These data indicated that the T-96-induced apoptosis was at least partially by modulating the expression of Bcl-2 family proteins.

### 3.3. T-96 Inhibits LSD1-Mediate Epigenetics Mechanism in SUM1315 Cells

Histone methylation, a covalent posttranslational modification (PTM) to histone proteins, plays a critical role in the regulation of chromatin structure, and its dynamics control several cellular processes such as proliferation, cell cycle, and programmed cell death [36–38]. Histone methyltransferases and demethylases are the major contributors to the establishment and maintenance of the level of different histone lysine methylations. So, they have been considered novel drug targets for cancer therapy [39]. LSD1, the first discovered histone lysine demethylase, is proposed to mediate the demethylation of H3K4me1/2 and H3K9me1/2 through a flavin adenine dinucleotide (FAD)-dependent amine oxidation reaction [40, 41]. LSD1 is highly expressed in various cancers and correlates with poor prognosis in patients [42, 43]. In breast cancer, LSD1 is highly expressed in ER-negative breast cancers and it has been proposed as a biomarker predicting aggressive biology [42].

**Figure 3:**
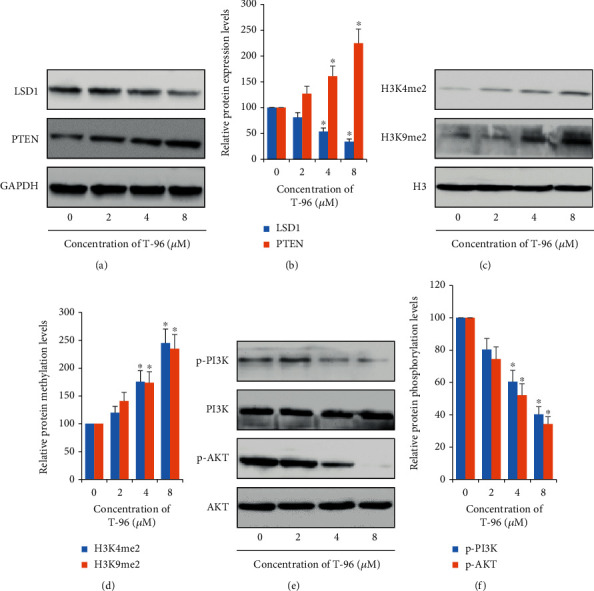
T-96 inhibited LSD1-mediate cell signal in the highly metastatic TNBC SUM-1315 cells. (a, b) T-96 inhibited LSD1 and its target protein expression by Western Blot; (c, d) LSD1 increased Histone H3K4 and Histone H3K9 methylation by Western Blot; (e, f) LSD1 inhibited PI3K/AKT cell signaling pathway. Values are statistically significant at ^∗^*P* < 0.05.

Figure 1(a) showed that TNBC cells were more sensitive than ER-positive breast cancer cells to the growth-inhibitory effects of T-96 and implied that T-96 played an antitumor effect via inhibition of LSD1 function. And Western Blotting assay was employed to prove this hypothesis. In Figures 3(a) and 3(b), the result showed that T-96 decreased the protein expression of LSD1. Next, we found that T-96 increased the protein expression of PTEN, which was directly regulated by LSD1 [44]. And T-96 also enhanced the methylation level of H3K4me2 in Figures 3(c) and 3(d). PTEN was a natural inhibitor of the PI3K/AKT cell signal pathway [45]. Figures 3(e) and 3(f) showed that T-96 repressed the PI3K/AKT cell signaling pathway activity.

**Figure 4:**
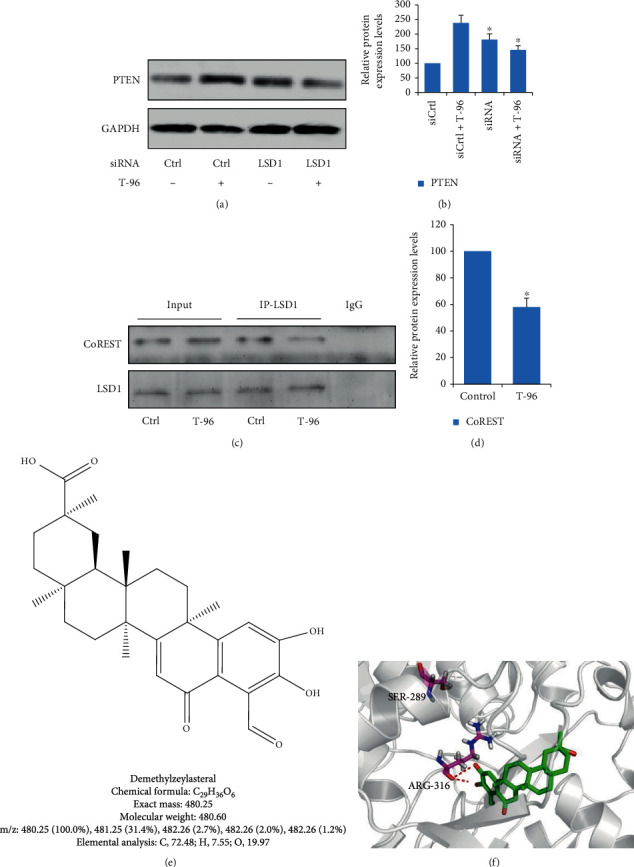
T-96 inhibited LSD1-mediate cell signal in the highly metastatic breast cancer cell SUM-1315. (a, b) LSD1 siRNA attenuated T-96 inhibited LSD1 pharmacological activity; (c, d) LSD1 inhibited CoREST/LSD1 protein-protein interaction by Co-IP assay. (e) Chemical structures of T-96; (f) the binding mode of T-96 bounded with the pocket of LSD1. Values are statistically significant at ^∗^*P* < 0.05.

To investigate whether the antitumor effect of T-96 was dependent on LSD1, we used siRNAs to knock down LSD1 expression, as shown in Figures 4(a) and 4(b), in LSD1-silenced SUM-1315 cells, the inhibitory effect of T-96 on PTEN protein expression was attenuated. Further results from the coIP assay confirmed that T-96 attenuated the interaction between LSD1 and CoREST in Figures 4(c) and 4(d). These results indicated that LSD1 was required for T-96-induced antitumor effects in SUM-1315 cells.

### 3.4. In Silico Mode of Action Prediction for T-96

We docked T-96 onto a published crystal structure of the LSD1 (Figures 4(e) and 4(f). The molecular docking predicted that the hydrogen bond formed between the phenolic hydroxy of T-96 and ARG316, which also contributed to the stable binding interaction in both complexes. This result indicated that T-96 processed powerful affinity toward LSD1 mainly through strong hydrogen bonding.

### 3.5. T-96-Induced antitumor Effects in Mice

**Figure 5:**
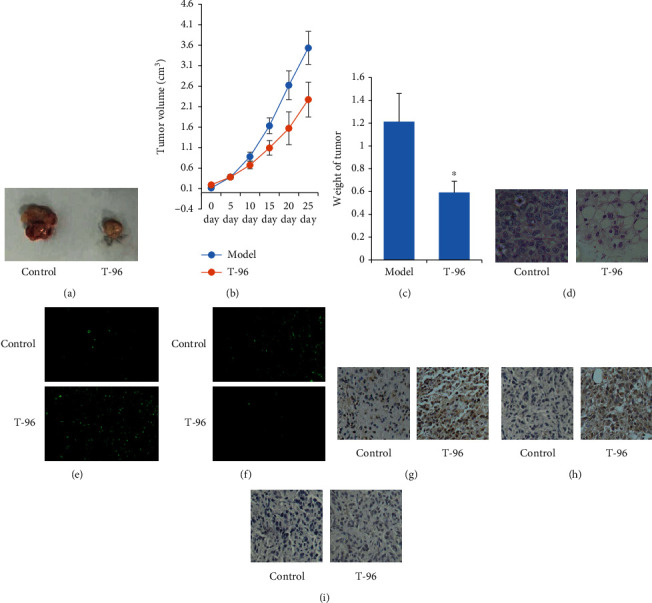
T-96-induced antitumor effects in mice. (a–d) T-96 inhibited SUM1315 cell proliferation *in vivo*; (e), T-96 induced SUM-1315 cells apoptosis by TUNEL; (f), T-96 decreased Ki-67 protein expression in SUM-1315 cells by immunofluorescence assay; (g and h) T-96 increased histone methylation by immunohistochemical assay; (i), T-96 increased PTEN protein expression in SUM-1315 cell by immunohistochemical assay. Values are statistically significant at ^∗^*P* < 0.05.

**Figure 6:**
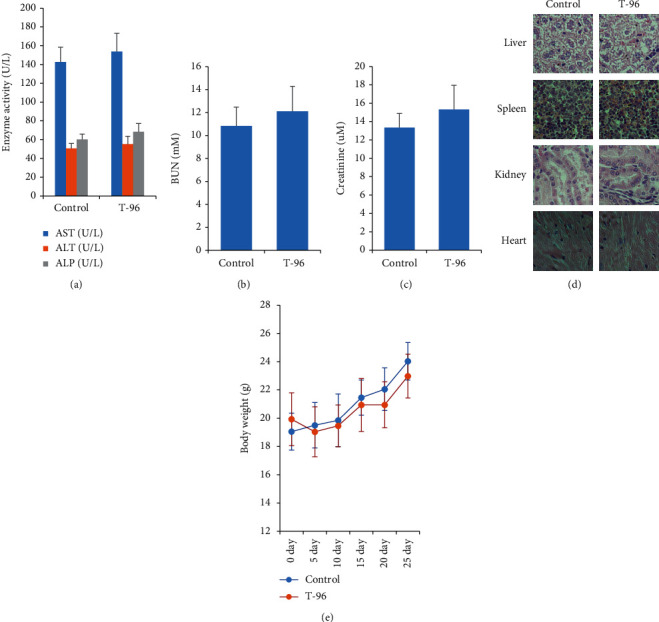
T-96 did not show significant organ toxicity in mice. (a), T-96 did not show significantly hepatotoxicity; (c, d) T-96 did not show significantly nephrotoxicity; (d), T-96 did not show significant organ toxicity by HE staining; (e), T-96 did not significantly reduce mouse body weight. Values are statistically significant at ^∗^*P* < 0.05.

Finally, we evaluated the antitumor efficacy of T-96 *in vivo*. In the xenograft model, SUM-1315 cells were inoculated subcutaneously into the BALB/c Nude mice. The mice were then treated by I.G with vehicle or T-96 (5 mg/kg/d) for 25 days. Compared with the vehicle-treated group, treatment with T-96 significantly decreased the growth of SUM-1315 xenograft (Figure 5(a)), reduced the volumes and weights of tumors (Figures 5(b) and 5(c)), and induced tumor death (Figure 5(d)).  Figure 5(e) showed that in TUNEL assay, compared with the control group, the green fluorescence emitted by the cells increased in the T-96 group. And T-96 decreased Ki-67 protein expression in SUM1315 cells by immunofluorescence assay (Figure 5(f)). These results suggested that T-96 induced cancer cell apoptosis and inhibited proliferation. T-96 increased the methylation levels of Histone H3K4me2 and H3k9me2 and enhanced the protein expression of PTEN by immunohistochemical staining assay (Figures 5(g)–5(i)). Further analysis revealed that T-96 did not show significant toxicity on the body weights and tissue (Figure 6). These results demonstrated that T-96 exhibited potent anti-tumor activity in vivo.

## 4. Discussion

TNBC is much more aggressive than others and leads to a poorer prognosis. So, developing a new therapy for metastatic breast cancer or TNBC is an appealing concept. SUM-1315 is a highly metastatic and TNBC cell line. So, in this study SUM-1315 was employed to evaluate the antitumor activity of T-96 and to investigate the underlying mechanisms *in vitro.* At the same time, the SUM-1315 xenograft model was used to evaluate the antitumor activity of T-96 *in vivo*.

Some articles have reported the inhibitory effect of T-96 on breast cancer and explored the mechanisms. ADP-ribosylation factor 1 (ARF1) plays a critical role in regulating vesicle formation and transport. The dysregulation of ARF1 expression and activity is involved in breast cancer. T-96 had the potential to inhibit ARF1 activity. T-96 could inhibit breast cancer proliferation via inhibiting hyperphosphorylation of pRB and its downstream pathway by targeting ARF1. [46] A previous study published by us found that T-96 could inhibit MDA-MB-231 (TNBC cells) migration, and suppress the expression of EMT-related genes and proteins. We found that the effect of T-96 inhibited invasion correlated with the mechanism by inhibiting both classical and nonclassical TGF-*β* signaling pathways [27]. However, in these previous studies, they did not investigate the antitumor effect and mechanisms of T-96 from the perspective of affecting methylation. As shown in Figure 1, we demonstrated that T-96 was an antitumor adjuvant agent, which had shown antitumor activity on breast cancer cell lines. And T-96 had a more robust activity against SUM-1315, which was a highly metastatic and TNBC cell line and was characterized by the lowest response to chemotherapies and the worst outcome [47]. We found that T-96 showed poor antitumor activity on MCF-7, which is another breast cell line, not a TNBC cell line. And, as reported in Figure 1(a) and 2(a), T-96 exhibited a dose-dependent antiproliferation effect against SUM-1315 cells. The members of the Bcl-2 family proteins are the hallmarks and regulators of the apoptotic process. Some Bcl-2 family members are located in the mitochondrial membrane, alter mitochondrial membrane permeability, trigger caspase activity, and determine the fate of the cells [45, 48]. And Bcl-2 family proteins are abnormally expressed in human breast cancer [49]. In SUM1315, T-96-induced apoptosis was associated with down-regulation of Bcl-2 and Bcl-xl expression, but upregulation of Bax expression in Figures 2(b) and 2(c).

PTEN, a kind of phosphatase, is found in almost all tissues of the human body. It modifies other proteins and fats (lipids) and removes phosphate groups from their substrates [50]. So, as a tumor suppressor, PTEN controls diverse cellular processes by protein posttranslational modifications. Some studies illustrated that there is tight crosstalk between PTEN and p53. PTEN could control the function of p53 by regulating p53 protein expression level and activity [45, 51]. Following, we detected the protein expression of PTEN, and found that T-96 significantly increased the amount of PTEN protein in Figures 3(a) and 3(b). PTEN could dephosphorylate phosphatidylinositol (3,4,5) trisphosphate, a product of PI3K, and inactivate PI3K/AKT cell signaling pathway to suppress cell survival [52–54]. So, PTEN is an antagonist of the PI3K/AKT signaling pathway. And we found that T-96 could also inhibit the phosphorylation of AKT (Figure 3(e), 3(f)). This result could further confirm the above observation.

LSD1, one member of the flavin-containing amine oxidase family and a part of transcriptional complexes, plays an important role in the regulation of transcription and gene expression [16, 55]. LSD1 is aberrantly overexpressed in a majority of cancers and has a significant correlation with aggressive pathological features and unfavorable prognosis [56–58]. Importantly, recent studies have illustrated inhibition of LSD1 activity or repression of LSD1 expression can inhibit tumor cell growth [59–62]. Many studies have focused on the role of LSD1 in breast cancer. They found that LSD1 was essential for breast cancer cell chemosensitivity, such as by coordinating with the SIN3A/HDAC complex and regulating a stem cell program [63, 64]. Moreover, LSD1 regulated ER*α* signaling in breast cancer, and inhibiting LSD1 induced significant growth arrest and apoptosis in the hormone-responsive breast cancer model [65]. And LSD1 activation promotes EMT programs in breast cancer [56]. So, most of these studies have focused on the inhibition of LSD1 to suppress invasion, metastasis, and EMT breast cancer [66–68]. Therefore, LSD1 is a therapeutic target in breast cancer therapy. In the present report, we found that T-96 could decrease the protein expression of LSD1 and increase the histone methylation of H3K4me2 and H3K9me2. Furthermore, T-96 could inhibit the protein expression of PTEN which was a direct target gene of LSD1 [44]. And knockdown of LSD1 gene expression by transfection of LSD1-specific siRNA could block the T-96-induced down-regulation of PTEN protein expression. CoREST, a functional corepressor required for the regulation of gene expression, could interact with LSD1 and enhance LSD1 demethylase activities toward H3K4 in vitro and in vivo [69, 70]. CoIP assay results showed that T-96 attenuated the interaction between LSD1 and CoREST. Moreover, the molecular docking assay further showed that T-96 processed powerful affinity toward LSD1 mainly interacting via hydrogen bonding. Altogether, these results indicated that restoration of LSD1 normal expression was necessary for T-96-induced antitumor activity.

**Figure 7:**
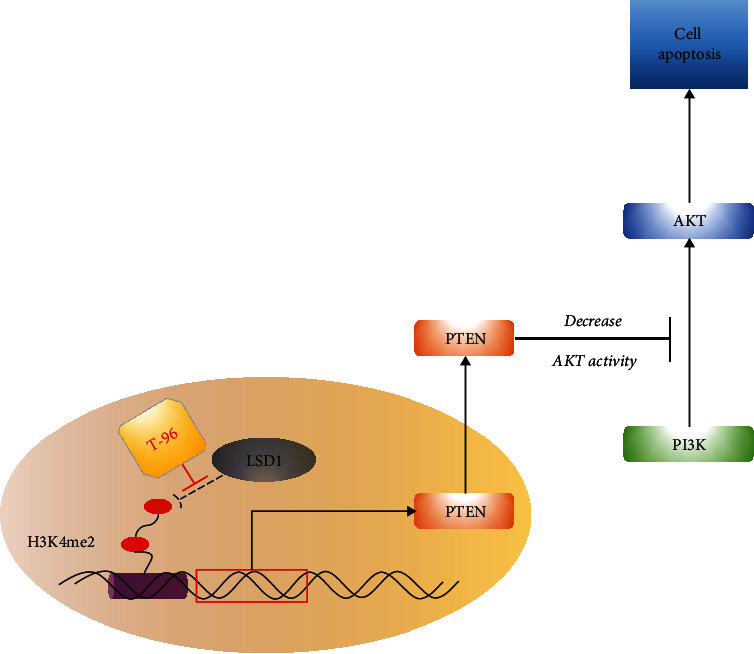
Antitumor effect of T-96 on breast cancer via LSD1-mediate epigenetic mechanisms.

Our data provide important information about the mechanisms of T-96 as a promising therapeutic agent, which effectively inhibited tumor cell growth and induced cancer cell apoptosis via LSD1-mediated epigenetic mechanisms. The specific mechanism of T-96 inducing tumor cells apoptosis is that T-96 could significantly decrease the protein expression of LSD1, increase its target protein PTEN expression and enhance histone methylation, and finally down-regulate the PI3K/AKT signaling pathway (Figure 7). Taken together, all these results demonstrate that LSD1 plays an important role in T-96-induced apoptosis in TNBC cells. Our findings suggest that T-96 deserves further investigation as a promising agent, because of the selective antitumor activity of T-96 to highly metastatic TNBC cells.

## Data Availability

The data used to support the findings of this study are included within the article. Further information is available from the corresponding author upon request.

## Abbreviations

T-96: Demethylzeylasteral
DMEM: Dulbecco's Modified Eagle's Medium
FBS: Fetal bovine serum
TUNEL: Terminal deoxynucleotidyl transferase dUTP nick end labeling
LSD1: Lysine (K)-specific demethylase 1A
coIP assay: Coimmunoprecipitation assay
MTT assay: 3-(4,5-cimethylthiazol-2-yl)-2,5-diphenyl tetrazolium bromide assay
TNBC: Triple-negative breast cancer
EMT: Epithelial-to-mesenchymal transition
TWHF: *Tripterygium wilfordii* Hook F.
